# Supplementary material for: Decidual Stromal Cell Ferroptosis Associated with Abnormal Iron Metabolism Is Implicated in the Pathogenesis of Recurrent Pregnancy Loss
Source: Int J Mol Sci. 2023 Apr 25;24(9):7836. doi: 10.3390/ijms24097836 (PMC10178458; doi:10.3390/ijms24097836)
Supplement: Supplementary file 1 [file ijms-24-07836-s001.zip › ijms-2278469-supplementary.pdf]

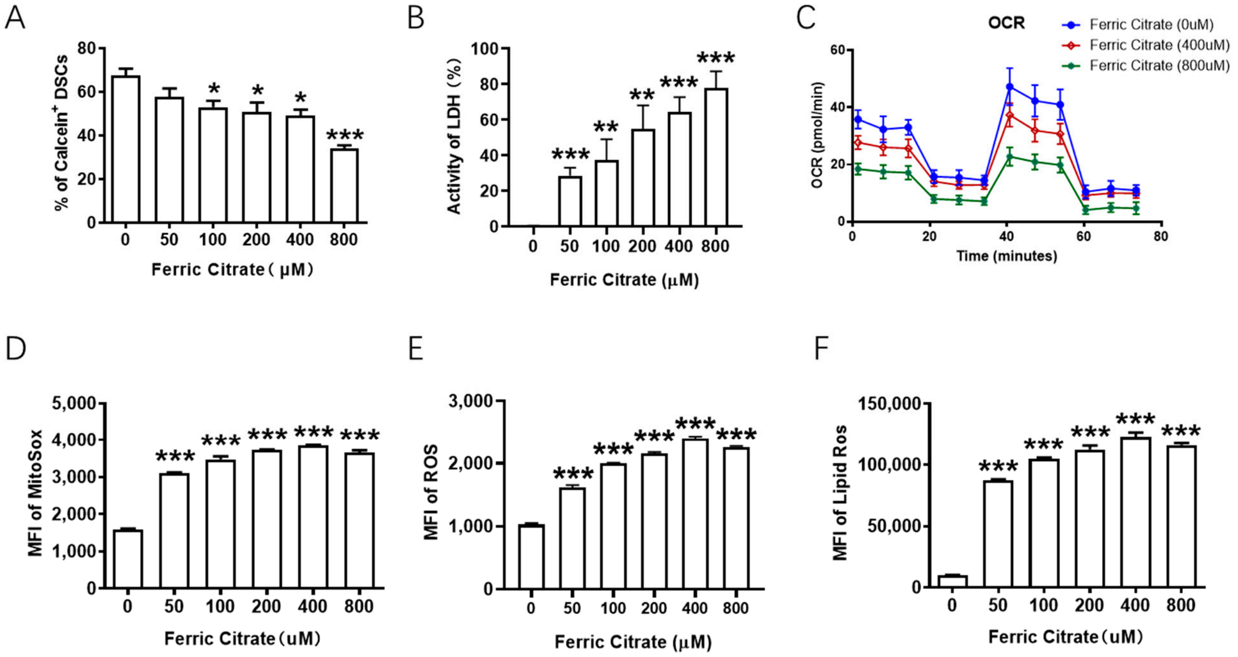

Figure S1. Ferric citrate caused DSC damages. (A) Quantification of flow cytometric analysis of calcein expression on DSCs stimulated with the indicated concentrations of ferric citrate. (B) Extracellular concentration of LDH was used to evaluate cell damage. (C) Oxygen Consumption Rate (OCR) of DSCs stimulated with the indicated concentrations of ferric citrate. (D) The levels of DSC mitochondrial superoxide were detected by flow cytometry. Summary of mean fluorescent intensity (MFI) from three independent experiments. (E) Intracellular ROS detected by flow cytometry. (F) The levels of intracellular lipid ROS of DSCs were detected by flow cytometry. Data represent the mean ± standard error of the mean (SEM) and are representative of three independent analyses. *$p<0.05$, **$p<0.01$, ***$p<0.001$.

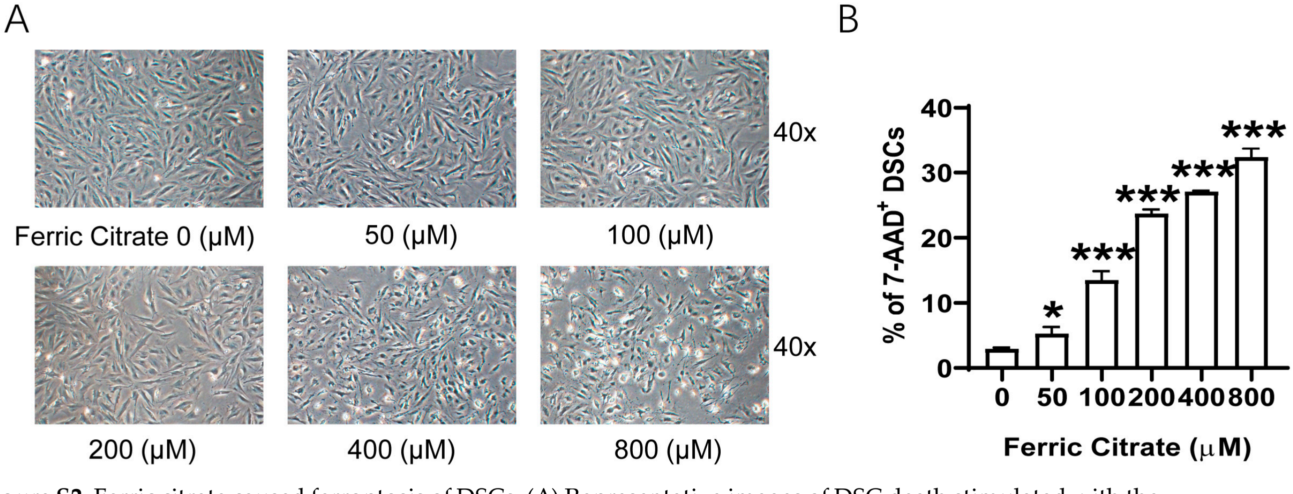

Figure S2. Ferric citrate caused ferroptosis of DSCs. (A) Representative images of DSC death stimulated with the indicated concentrations of ferric citrate were observed by Inverted Phase Contrast Microscope. (B) Cell death was evaluated based on 7-AAD expression on DSCs stimulated with the indicated concentrations of ferric citrate by flow cytometry. Data represent the mean ± SEM and are representative of three independent analyses. *$p<0.05$, ***$p<0.001$.
